# Clean energy consumption, sleep duration, and the association of cancer: findings from the China health and retirement longitudinal study

**DOI:** 10.3389/fonc.2024.1327257

**Published:** 2024-04-18

**Authors:** Jushuang Li, Yutong Han, Wendi Bai

**Affiliations:** ^1^ Department of Medical Statistics, School of Public Health, Sun Yat-Sen University, Guangzhou, Guangdong, China; ^2^ Sun Yat-Sen Global Health Institute, Institute of State Governance, Sun Yat-Sen University, Guangzhou, Guangdong, China; ^3^ Department of Epidemiology, School of Public Health, Fudan University, Shanghai, China; ^4^ Department of Public Health, Guangzhou Nansha Center for Disease Control and Prevention, Guangzhou, China

**Keywords:** clean energy consumption, sleep duration, cancer, LOESS, CHARLS

## Abstract

**Objective:**

Studies of the association between clean energy consumption, sleep duration, and cancer are still extremely limited. We aim to investigate the individual or joint role of clean energy consumption, and sleep duration in cancer onset.

**Methods:**

We used data from the China Health and Retirement Longitudinal Study. Multivariable locally weighted regression (LOESS) models were first used to assess the individual association of daily sleep time with the risk to develop cancer. Multivariate logistic regression models were conducted on the individual and interaction effects of daily sleep time and cooking fuel.

**Results:**

We found that short sleep duration (≤6 hours) and non-clean energy consumption were respectively associated with an increased risk of cancer among older Chinese(p<0.05). We assessed daily sleep time in four quartiles, the adjusted odds ratios (AOR), and 95% confidence intervals (95% CI) for participants in the second (5.0 to <6.5 hours), third (6.5 to <8.0 hours), and fourth quartiles (≥8.0 hours) were 0.88 (95% CI: 0.65-1.20), 0.61 (95% CI: 0.40-0.91), and 0.53 (95% CI: 0.37-0.77), respectively. When we set the cutoff point(6.5 hours), participants who slept more than 6 hours had a 39% lower risk of cancer (AOR: 0.61, 95% CI: 0.46-0.79) compared with others. On the other hand, we conducted that exposure to clean fuel from cooking was negatively associated with cancer incidence (AOR: 0.73, 95% CI: 0.54, 0.97). Furthermore, the combination of longer sleep and cleaner fuels showed the lowest OR for cancer (AOR: 0.39, 95% CI: 0.24, 0.65).

**Conclusion:**

Our study showed that sleep duration and clean energy consumption were significantly associated with cancer in elderly Chinese people. In addition, the prevalence of cancer was higher among people who slept less than six hours and used non-clean energy sources. Paying greater attention to the effects of sleep duration and clean energy on the risk of cancer may yield practical implications for cancer prevention.

## 1 Introduction

Cancer is the second leading cause of death, years of life lost (YLLs), and disability-adjusted life years (DALYs) behind cardiovascular diseases. In 2019, there were 23.6 million new cancer cases and 10 million cancer-related deaths, posing a significant threat to human life and well-being. China, the biggest developing country in the world, has an immense cancer burden. Cancer has emerged as a critical public health concern, posing a significant threat to human life and well-being (1). In 2020, China witnessed a staggering number of 4,568,754 new cases of cancer, leading to 3,002,899 deaths, accounting for 30.2% of all cancer-related deaths worldwide (2). Despite an overall improvement of approximately 10% in the survival rate of malignant tumors across China, the burden of cancer continues to rise (3). The substantial changes in cancer burden observed over the past few decades can be attributed primarily to significant improvements in living conditions, which increases average life expectancy of the population and tends to age- A known risk factor for cancer, particularly in rural China. While the incidence rate of cancer in urban areas slightly surpasses that of rural areas, the mortality rate in rural regions is slightly higher than in urban counterparts. It is imperative, therefore, for governments and relevant agencies to continuously update policies and strategies to effectively prevent cancer.

Household Air Pollution (HAP) is a prominent environmental risk factor. HAP arises from the incomplete combustion of solid fuels used in households, including coal, biomass, and others. These fuels generate a range of pollutants, such as particulate matter, sulfur dioxide, carbon monoxide, nitrogen dioxide, formaldehyde, and heavy metals (4). In the period from 2001 to 2010, the Chinese government devoted considerable efforts to enhancing access to clean fuels by implementing measures such as economic subsidies (5). Despite the implementation of various measures, the contamination caused by gases remains a significant concern for approximately 91% of the global population, as reported by the World Health Organization. Developing economies, where a significant proportion of the population resides in rural areas, are particularly affected by this issue. As of 2019, more than 60% of the rural population in China continued to lack access to clean fuels, while worldwide, an astounding 2.8 billion people were in the same situation (6). The reliance on solid fuels, such as coal, wood, and crop residues, for cooking and heating is widespread in these regions. Additionally, rural areas typically accommodate larger populations compared to urban areas. Therefore, prioritizing the reduction of HAP exposure and the promotion of clean energy utilization in rural settings is of utmost importance for achieving public health objectives and alleviating the global disease burden.

Several previous studies have suggested a link between sleep duration and cancer, highlighting the importance of sleep for physical and mental health (7–9). Sleep duration have also been associated with the development of other diseases (10–12). Despite this, few studies have evaluated the effects of sleep duration and clean energy consumption on cancer risk in the general population. To address this gap, we conducted a cross-sectional study that examined the effects of sleep duration and clean energy consumption on cancer risk. Our study drew upon data from the China Longitudinal Study of Health and Retirement (CHARLS) in 450 Chinese communities. Through our analysis, we aimed to shed light on the potential independent and synergistic effects of these two factors, which could help inform policy and public health interventions aimed at reducing cancer risk in the general population.

## 2 Methods

### 2.1 Data source and study population

This study is based on data from the fourth edition of the China Health and Retirement Longitudinal Study (CHARLS 2018), which was initiated in 2011 by the National Development Institute at Peking University. The data collection utilized a multistage probability sampling method to ensure a nationally representative sample of Chinese adults aged 45 and above. The study covered 150 prefectures and 450 villages in China. Micro survey data were collected from 10,000 households and 170,000 individuals, and follow-up visits were conducted every two years by trained interviewers or healthcare professionals. The data collection involved one-to-one interviews to increase response rates. The questionnaire covered a wide range of topics, including social, economic, and health-related issues. Follow-up questions were tailored to the participants’ age and social development, based on the life cycle theory, to ensure the scientific and innovative nature of the data. In the survey, 19,816 respondents were asked if they had been informed by a physician about 14 chronic conditions, including cancer. Among these respondents, 449 reported a history of cancer. After removing missing values and errors, a final sample of 13,147 middle-aged and older individuals aged 45 and above was used for this study, including 271 individuals with a history of cancer.

### 2.2 Outcome

During the survey, recording the cancer incidence was self-report. All participants were asked the following question: “Have you ever been diagnosed with cancer or malignant tumor (excluding minor skin cancers) by a doctor?” Participants who responded affirmatively were then asked about the location of their cancer, including the primary site and any metastasis. They were provided with a list of options and instructed to circle all applicable locations, such as brain, thyroid, lung, breast, stomach, liver or other location. Participants who reported having cancer, either through self-report or by listing cancer as the cause of death if they passed away during the study period, were classified as having incident cancer. Furthermore, participants who died during the study period and with cancer listed as the cause of death were also identified as having incident cancer.

### 2.3 Exposure and covariates

According to the questionnaire, cooking fuel was divided into the following: (1) coal; (2) natural gas; (3) marsh gas; (4) liquefied petroleum gas; (5) electric; (6) crop residue/wood burning; and (7) others. The interviewer asked the question of “What is the main fuel used for cooking in your home?” If the respondent answered clean fuel (natural gas, marsh gas, liquefied petroleum gas, electric), we set CHE (Clean Household Energy) = 1; if the respondent answered solid fuel (coal or crop residue/wood burning), we set CHE = 0.

Sleep duration was assessed by asking subjects the following questions: “During the past month, how many hours of actual sleep did you get at night (average hours for one night)? (This may be shorter than the number of hours you spend in bed.)”

Considering that residents’ health may be affected by multi-dimensional factors, in order to increase the explanatory power of the model, factors affecting residents’ well-being will be more comprehensive and complete. The following factors were selected as potential confounding variables: age; gender (female vs. male); marital status (married vs. divorced/separated/widowed/never married); education level (illiterate and primary school, middle school, high school or college and above); residence (rural vs. urban) and alcohol consumption (no vs. yes).

### 2.4 Statistical analyses

Population characteristics between the cancer and non-cancer group were evaluated as follows. Continuous variables not met normal distribution were descripted with median (1st quartile, 3rd quartile) and compared with Mann-Whitney U test. Chi-square tests were used to compare the differences of the proportion of categorical variables between the two groups. To thoroughly examine the relationship between cancer and daily sleep time, multivariable locally weighted regression (LOESS) models were firstly used to assess the individual association of daily sleep time with the risk to develop cancer. The individual and interaction effects of daily sleep time and cooking fuel were determined using multivariate logistic regression models. The covariates included in the multivariate model analysis are based on the literature and variables with a P-value less than 0.1 in the univariate analysis. Data management, analysis and figure drawing were finished using R version 4.3.0 (Copyright ^©^ 2023 The R Foundation for Statistical Computing). *p*<=0.05 was set as significant level and all tests were 2-sides.

## 3 Result

### 3.1 Baseline characteristics of study population

**Table 1** presents the general characteristics of the study population and provides a comparison of demographic data for the cases (cancer group) and controls (non-cancer group). Among the cases, the median age (first and third quartiles) was 65.0 (59.0 and 71.0) years, and 41.0% were males. In the controls group, the median age was 64.0 (57.0 and 71.0) years, and 46.7% were males. The prevalence of cancer in the overall study population was 2.44%.

**Table 1:** Characteristics of the study population in two groups in CHARLS.

Characteristics	Control (n=12876)	Cases (n= 271)	*P* value
Age, years,	64.0 (57.0,71.0)	65.0 (59.0,71.0)	0.030
Sex, n (%)			0.061
male	6012 (46.7)	111 (41.0)	
female	6864 (53.3)	160 (59.0)	
Marital status, n (%)			0.462
Partnered	10661 (82.8)	229 (84.5)	
Single	2215 (17.2)	42 (15.5)	
Education, n (%)			0.026
Below primary school	8843 (68.7)	191 (70.5)	
Primary school	2581 (20.0)	48 (17.7)	
Middle school	1018 (7.9)	15 (5.5)	
High school and above	434 (3.4)	17 (6.3)	
Residence, n (%)			<0.001
Central of City/Town	2230 (17.3)	71 (26.2)	
Urban-Rural Integration Zone	856 (6.6)	24 (8.9)	
Rural	9736 (75.6)	175 (64.6)	
Special Zone	54 (0.4)	1 (0.4)	
Alcohol consumption, n (%)			0.037
Yes	10220 (79.4)	201 (74.2)	
No	2656 (20.6)	70 (25.8)	
Clean energy, n (%)			0.009
No	8433 (65.5)	198 (73.1)	
Yes	4443 (34.5)	73 (26.9)	
Number of chronic diseases, n	0.0 (0.0,1.0)	1.0 (1.0,2.0)	<0.001
Sleep duration, hours	6.0 (5.0,8.0)	6.0 (4.0,7.0)	<0.001
Water quality			0.056
Tap water	10328 (80.2)	230 (84.9)	
Non-tap water	2548 (19.8)	41 (15.1)	
Air quality, people’s satisfaction with indoor air quality			0.175
Not at all satisfied	588 (4.6)	11 (4.1)	
Not very satisfied	3323 (25.8)	54 (19.9)	
Somewhat satisfied	7077 (55.0)	160 (59.0)	
Very satisfied	1494 (11.6)	34 (12.5)	
Completely satisfied	394 (3.1)	12 (4.4)	

### 3.2 Association between the risk of cancer and sleep duration or clean energy usage

**Figure 1:**
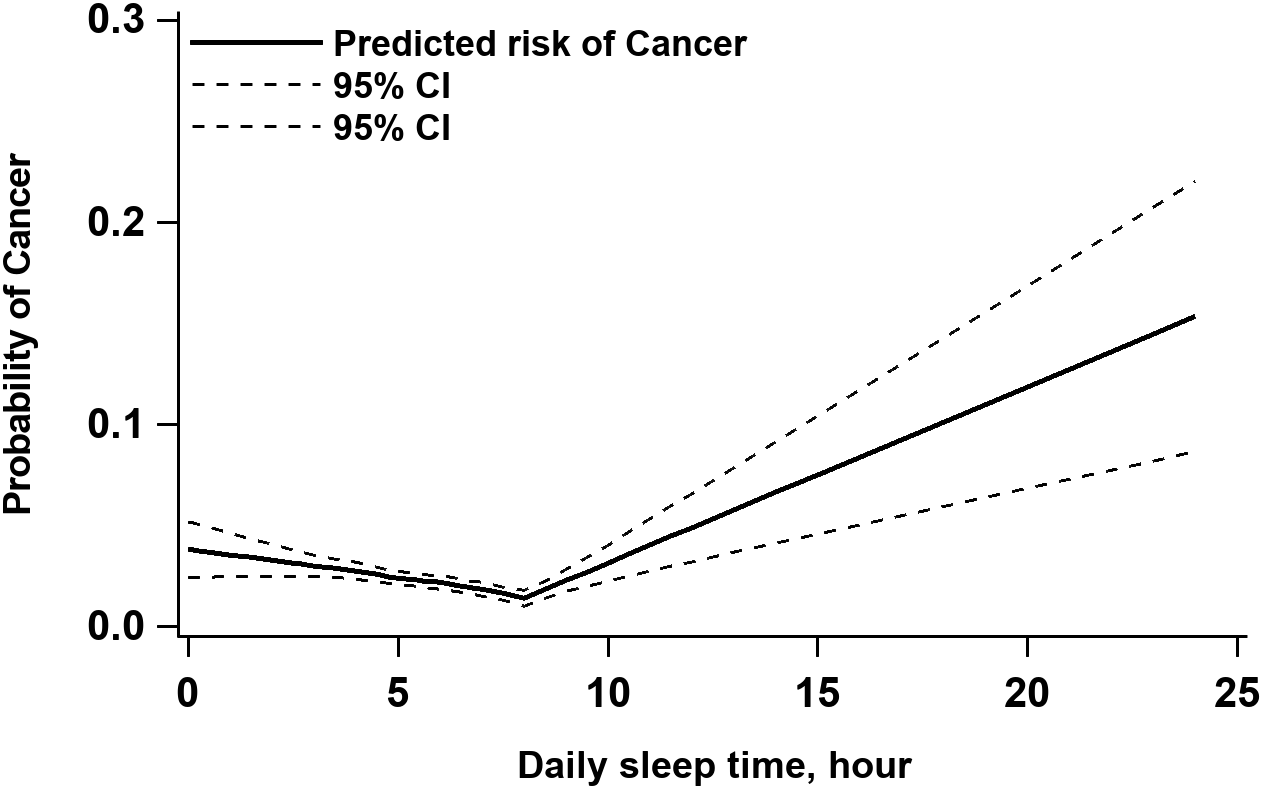
Association between sleep duration and cancer prevalence based on multivariable locally weighted regression models.

Overall, daily sleep time was significantly negatively associated with the risk of cancer when it was less than 8 hours, and positively associated when it was greater than 8 hours (**Figure 1**). As shown in **Table 2**, when daily sleep time was assessed in quartiles, the adjusted odds ratios (AOR) and 95% confidence intervals (95% CI) for participants in the second (5.0 to <6.5 hours), third (6.5 to <8.0 hours), and fourth quartiles (≥8.0 hours) were 0.88 (95% CI: 0.65-1.20), 0.61 (95% CI: 0.40-0.91), and 0.53 (95% CI: 0.37-0.77), respectively, in comparison with those in the first quartile (*P* for trend through the quartiles < 0.001). When we used the median daily sleep duration as the cutoff point, older adults who slept longer had a 39% lower risk of cancer (AOR: 0.61, 95% CI: 0.46-0.79) compared with those who slept less than 6 hours.

**Table 2:** Association between sleep duration and cancer prevalence based on univariate and multivariate logistic regression models.

Daily sleep time	Total	Cases (%)	OR (95%CI)	*P* value	AOR (95%CI)	*P* value
Daily sleep time, hours						
0~	2777	81 (2.90)	1.00 (1.00,1.00)	Ref.	1.00 (1.00,1.00)	Ref.
5.0~	4544	107 (2.40)	0.80 (0.60,1.08)	0.141	0.88 (0.65,1.19)	0.410
6.5~	2310	37 (1.60)	0.54 (0.37,0.80)	0.002	0.61 (0.41,0.91)	0.016
8.0~24.0	3516	46 (1.30)	0.44 (0.31,0.64)	<0.001	0.53 (0.37,0.78)	0.001
Trend test				<0.001		<0.001
Daily sleep time ≤ 6 hours						
Yes	7321	188 (2.60)	1.00 (1.00,1.00)	Ref.	1.00 (1.00,1.00)	Ref.
No	5826	83 (1.40)	0.55 (0.42,0.71)	<0.001	0.61 (0.47,0.79)	<0.001

OR, Odds Ratio; AOR, Adjusted Odds Ratio; 95% CI, 95% Confidence interval.

Adjusted factors:age, sex, Marital status, Education, Residence, Alcohol consumption, Clean energy, Number of chronic diseases, Air quality, Water quality.

**Table 3** presents the risk of cancer associated with household clean fuel. A statistically significant association was observed between clean fuel exposure from cooking (*p* = 0.032). Exposure to clean fuel from cooking was negatively associated with cancer incidence (AOR: 0.73, 95% CI: 0.54, 0.97) for cooking.

**Table 3:** Association between clean energy usage and cancer prevalence based on univariate analysis.

Clean energy consumption	Total	Cases,n(%)	OR (95%CI)	*p* value	AOR (95%CI)	*p* value
No	8631	198 (2.30)	1.00 (1.00,1.00)	Ref.	1.00 (1.00,1.00)	Ref.
Yes	4516	73 (1.60)	0.70 (0.53,0.92)	0.010	0.76 (0.56,1.02)	0.066

OR, Odds Ratio; AOR, Adjusted Odds Ratio; 95% CI, 95% Confidence interval.

Adjusted factors:age, sex, Marital status, Education, Residence, Alcohol consumption, Clean energy, Number of chronic diseases, Air quality, Water quality.

### 3.3 Joint association between sleep duration and clean energy usage with cancer

**Table 4** illustrated the interaction effect between sleep duration and clean energy on cancer prevalence. The proportions of cancer among participants in different categories were as follows: 2.80% for those with lower daily sleep time and unclean energy usage (category A), 2.10% for lower daily sleep time and clean energy usage (category B), 1.70% for higher daily sleep time and unclean energy usage (category C), and 1.00% for higher daily sleep time and clean energy usage (category D). Notably, a significant reduction in the probability of cancer was observed across these four categories.

**Table 4:** Joint association between sleep duration and clean energy usage with cancer prevalence.

Daily sleep time ≤ 6 hours	Clean energy	n	Cases (%)	OR (95%CI)	*p* value	AOR (95%CI)	*p* value
Yes	No	4777	134 (2.80)	1.00 (1.00,1.00)	Ref.	1.00 (1.00,1.00)	Ref.
Yes	Yes	2544	54 (2.10)	0.75 (0.55,1.03)	0.080	0.82 (0.58,1.15)	0.247
No	No	3854	64 (1.70)	0.59 (0.43,0.79)	<0.001	0.65 (0.48,0.88)	0.006
No	Yes	1972	19 (1.00)	0.34 (0.21,0.55)	<0.001	0.41 (0.25,0.68)	<0.001
Interaction					0.391		0.420

OR, Odds Ratio; AOR, Adjusted Odds Ratio; 95% CI, 95% Confidence interval.

Adjusted factors:age, sex, Marital status, Education, Residence, Alcohol consumption, Clean energy, Number of chronic diseases, Air quality, Water quality.

When compared to category A, the adjusted odds ratios (AOR) (95% CI) for participants in categories B, C, and D were 0.78 (0.56 to 1.10), 0.64 (0.48 to 0.87), and 0.39 (0.24 to 0.65), respectively. This implies that subjects with higher daily sleep time and clean energy usage had the lowest odds of cancer, which decreased significantly by 61% after accounting for potential confounding factors. Therefore, the joint association between daily sleep time and clean energy usage is linked to cancer risk, although no statistically significant interaction was observed (*p*=0.444).

**Table 5** shows the classification of specific cancers in relation to sleep duration and the use of clean energy. And the analysis revealed that patients with lung cancer and breast cancer were more likely to sleep for less than 6 hours and use non-clean fuels (*p*<0.05).

**Table 5:** Association of different sites cancer with daily sleep time and clean energy usage.

Variables	Total number	Daily sleep time ≤ 6 hours and unclean energy	Daily sleep time ≤6 hours and clean energy	Daily sleep time >6 hours and unclean energy	Daily sleep time>6 hours and clean energy	*p* value
Brain	12	5	3	4	0	0.540
Thyroid	10	6	2	2	0	0.342
Lung	33	20	5	5	3	0.034
Breast	39	23	6	7	3	0.030
Stomach	24	10	8	5	1	0.165
Liver	18	9	4	4	1	0.497
Cervix	24	12	4	7	1	0.361
Endometrium	24	10	4	7	3	0.945
Colon or rectum	26	14	4	6	2	0.298
Other organ	41	15	12	11	3	0.284

## 4 Discussion

Our study, which included a sizable and representative sample of the general population, has demonstrated that both reduced sleep duration and the use of non-clean energy are significant contributors to an elevated risk of cancer, even after adjusting for various known risk factors. Therefore, cancer surveillance and monitoring should be enhanced for older adults who use non-clean energy sources and have shortened sleep schedules. Moreover, public health interventions that promote the use of clean energy and adequate sleep may represent an effective strategy to reduce cancer risk in the general population.

Previous studies have shown that the incidence of cancer and the level of air pollutants have increased significantly, including but not limited to lung cancer, breast cancer, etc. Adhikari A. and others found that in some sub-Saharan countries, the incidence of cancer and the level of air pollutants have increased significantly in the past 15 years (13). Cooking and indoor incineration have been identified as significant risk factors for lung cancer, especially among women (14). Qing Lan and others reviewed a series of case-control and cohort studies conducted in Xuanwei County, Yunnan Province, China, which have linked household air pollution exposure to the high incidence of lung cancer. They found that the risk of cancer in users of smoky coal was higher than that in users of smokeless coal (15). Jon G. Ayres and others quantified the impact of biomass fuel and coal use on lung cancer, and their research findings indicate that burning coal and biomass in the home is consistently associated with an increased risk of lung cancer. Moreover, the combined effect estimate of coal smoke as a lung carcinogen (OR 1.82, 95% CI 1.60-2.06) is greater than that of biomass smoke (OR 1.50, 95% CI 1.17-1.94). The risk of lung cancer in women (OR 1.81, 95% CI 1.54-2.12) is higher than that in men (OR 1.16, 95% CI 0.79-1.69) (16). Prospective cohort studies conducted in the United States and Puerto Rico have also indicated a potential association between the use of wood-burning stoves and fireplaces and an increased risk of breast cancer (HR = 1.11, 95% CI: 1.01-1.22) (17). Wangsheng Feng and others found that long-term solid fuel combustion may increase the risk of breast cancer, and the association strength of coal users is greater than that of wood users (18).

In recent years, there has been an emerging interest in exploring the potential link between cancer and sleep duration. A meta-analysis has revealed a positive association between both very short (4-5 hours) and very long (over 8 hours) sleep duration and overall cancer mortality in the general population (19). Another meta-analysis found that short sleep duration increases the risk of cancer, while long sleep duration increases the risk of cancer in Asians. However, these findings have not consistently been supported in dose-response meta-analyses (12).

Emmanuel Stamatakis, in their exploration of the impact of sleep and physical activity (PA) on health, found that compared with the high PA-healthy sleep group (reference), the no moderate-to-vigorous PA-poor sleep group had the highest mortality risks for total cancer (1.45 (1.18 to 1.77)) and lung cancer (1.91 (1.30 to 2.81)) (20). In addition, Paolo Boffetta found that long sleep was associated with gastric cancer, its subsites, and histological types (21). However, unlike cancers caused by air pollutants, it has not been found that the length of sleep affects the incidence of breast cancer. Angel T. Y. Wong. and Jia He, in their respective meta-analysis studies, found little or no effect of sleep duration on breast cancer risk (22, 23).

Building upon these studies, our research aimed to assess the combined effects of short sleep duration and non-clean energy use on cancer risk among middle-aged and older individuals in China. By investigating the interaction between these two risk factors, we sought to provide valuable insights into the intricate interplay of environmental and lifestyle elements contributing to cancer risk.

To the best of our knowledge, there is a scarcity of studies examining the relationship between sleep duration, clean fuel use, and cancer risk in a representative population in China. This research gap is of great importance due to the escalating incidence and mortality rates of cancer in the country, which have been rising at rates of 2.5% to 3.9% annually (24). In the year 2020 alone, China reported a staggering 4,569,000 new cancer cases and 3.03 million cancer-related deaths, solidifying its position as the country with the highest incidence and mortality rates of cancer worldwide (25).

Our study findings provide compelling evidence that the combined impact of short sleep duration and non-clean energy use may heighten the risk of developing cancer among middle-aged and elderly individuals in China. The exact biological mechanisms underlying the carcinogenic effects of air pollution are not yet fully elucidated. However, household air pollution (HAP) consists of various mutagens and carcinogens, including sulfur-containing compounds (such as SO3 and H2SO4), polycyclic aromatic hydrocarbons (such as benzo(a)pyrene and polar compounds), among others (26, 27). Previous research has demonstrated a strong association between the presence of carcinogen DNA adducts and cancer risk (28–30). However, the ability of an individual’s repair mechanisms to eliminate these DNA adducts plays a crucial role in determining whether they lead to DNA mutations and subsequent carcinogenesis (31).

Oxidative stress (OS) is a crucial mechanism underlying the carcinogenic effects of air pollution (32). OS occurs when there is an accumulation of free radicals, including reactive oxygen species (ROS) and reactive nitrogen species (RNs). Nitrogen oxides (NO and nitrogen dioxide) and metals present in household air pollution (HAP) can increase the levels of intracellular free radicals (33). Early studies have demonstrated that exposure of mouse fibroblasts to ROS can lead to the oncogenic transformation of cells (34). Additionally, ROS have been shown to promote cell proliferation, invasiveness, angiogenesis, and metastasis, while inhibiting apoptosis, thus acting as potential tumor-promoting factors (35). Furthermore, air pollution compounds can induce the release of proinflammatory cytokines, such as TNF-α, IL-6, and granulocyte-macrophage colony-stimulating factor (GM-CSF), leading to low-grade chronic inflammation in the airways and the entire body (36, 37).

On the other hand, reduced sleep duration has been linked to metabolic alterations, which can result in increased release of C-reactive protein, interleukin-6 (IL-6), and tumor necrosis factor (TNF). These cytokines have the potential to activate nuclear factor-kappa B (NF-κB) (38). It is conceivable that sleep disturbances and exposure to air pollution may amplify the risk of cancer through this pathway. Our hypothesis suggests that the simultaneous presence of short sleep duration and non-clean energy use may accelerate cancer progression to a greater extent than when they occur separately. Nevertheless, further evidence from experimental studies is required to validate this hypothesis in future research.

Our study has several strengths that contribute to its significance. Firstly, our study includes a nationally representative population, which enhances the generalizability of our findings. Secondly, we have identified an association between the combined effects of sleep duration and clean energy use and the risk of cancer among middle-aged and elderly individuals in China. However, despite these strengths, our study also has some limitations that should be acknowledged. Firstly, recording the cancer incidence was self-report, which may introduce recall bias. However, a previous prospective cohort study that compared self-reported cancer data with the National Cancer Registry reported high validity, with high sensitivity (0.93) and specificity (0.98) (39). Secondly, the cross-sectional design of our study prevents the establishment of causality between sleep duration, clean energy use, and cancer incidence. Future studies should consider a prospective design to better establish temporal relationships and causality. Finally, we lacked information on important factors such as cancer age at diagnosis, stage, and treatment regimen, which may have influenced the health status of cancer survivors included in our study. These factors should be considered in future studies to provide a more comprehensive understanding of the associations observed. In conclusion, future research should consider prospective designs with objective measures of sleep duration, air pollution exposure, and cancer outcomes to establish causality and provide more robust evidence to inform prevention and control efforts.

## 5 Conclusion

In conclusion, our study has provided evidence suggesting that the combination of short sleep duration and non-clean fuel use is associated with an elevated risk of overall cancer among middle-aged and elderly individuals in China. These findings emphasize the significance of addressing both sleep and clean energy in cancer management. Healthcare professionals should advise patients to prioritize sufficient sleep and the use of clean energy sources as part of comprehensive strategies for cancer prevention.

## Data availability statement

The original contributions presented in the study are included in the article/supplementary materials. Further inquiries can be directed to the corresponding author.

## Ethics statement

The study was carried out in accordance with the principles outlined in the Declaration of Helsinki, and received approval from the Ethics Committee of Peking University (IRB00001052‐11015). Approval for data collection was obtained from the Ethical Review Committee at Peking University, which is renewed on a yearly basis. All participants involved in the study have provided written informed consent.

## Author contributions

WB: Data curation, Supervision, Validation, Writing – review & editing. JL: Methodology, Project administration, Software, Writing – original draft. YH: Formal analysis, Visualization, Writing – review & editing.
